# The effects of phosphocreatine disodium salts plus blueberry extract supplementation on muscular strength, power, and endurance

**DOI:** 10.1186/s12970-021-00456-y

**Published:** 2021-09-09

**Authors:** John Paul V. Anders, Tyler J. Neltner, Robert W. Smith, Joshua L. Keller, Terry J. Housh, F. Joseph Daugherty, Michael S. Tempesta, Alekha K. Dash, Daniel J. Munt, Richard J. Schmidt, Glen O. Johnson

**Affiliations:** 1 Department of Nutrition and Human Sciences, University of Nebraska-Lincoln, Lincoln, NE 68510 USA; 2 Department of Health, Kinesiology and Sport, University of South Alabama, Mobile, AL 36688 USA; 3 Phenolics LLC, Omaha, NE 68144 USA; 4 Department of Pharmacy Sciences, School of Pharmacy and Health Professions, Omaha, NE 68178 USA

**Keywords:** Phosphocreatine, Blueberry, Polyphenols, Creatine monohydrate, Antioxidant, Isokinetic, Strength, Power, Muscular endurance

## Abstract

**Background:**

Numerous studies have demonstrated the efficacy of creatine supplementation for improvements in exercise performance. Few studies, however, have examined the effects of phosphocreatine supplementation on exercise performance. Furthermore, while polyphenols have antioxidant and anti-inflammatory properties, little is known regarding the influence of polyphenol supplementation on muscular strength, power, and endurance. Thus, the purpose of the present study was to compare the effects of 28 days of supplementation with phosphocreatine disodium salts plus blueberry extract (PCDSB), creatine monohydrate (CM), and placebo on measures of muscular strength, power, and endurance.

**Methods:**

Thirty-three men were randomly assigned to consume either PCDSB, CM, or placebo for 28 days. Peak torque (PT), average power (AP), and percent decline for peak torque (PT%) and average power (AP%) were assessed from a fatigue test consisting of 50 maximal, unilateral, isokinetic leg extensions at 180°·s^− 1^ before and after the 28 days of supplementation. Individual responses were assessed to examine the proportion of subjects that exceeded a minimal important difference (MID).

**Results:**

The results demonstrated significant (*p* < 0.05) improvements in PT for the PCDSB and CM groups from pre- (99.90 ± 22.47 N·m and 99.95 ± 22.50 N·m, respectively) to post-supplementation (119.22 ± 29.87 N·m and 111.97 ± 24.50 N·m, respectively), but no significant (*p* = 0.112) change for the placebo group. The PCDSB and CM groups also exhibited significant improvements in AP from pre- (140.18 ± 32.08 W and 143.42 ± 33.84 W, respectively) to post-supplementation (170.12 ± 42.68 W and 159.78 ± 31.20 W, respectively), but no significant (*p* = 0.279) change for the placebo group. A significantly (*p* < 0.05) greater proportion of subjects in the PCDSB group exceeded the MID for PT compared to the placebo group, but there were no significant (*p* > 0.05) differences in the proportion of subjects exceeding the MID between the CM and placebo groups or between the CM and PCDSB groups.

**Conclusions:**

These findings indicated that for the group mean responses, 28 days of supplementation with both PCDSB and CM resulted in increases in PT and AP. The PCDSB, however, may have an advantage over CM when compared to the placebo group for the proportion of individuals that respond favorably to supplementation with meaningful increases in muscular strength.

## Background

Creatine is a naturally occurring, nitrogenous compound synthesized from arginine, glycine, and methionine that can be produced endogenously by the liver, kidneys, and pancreas, or obtained exogenously from dietary sources such as red meats and fish [1–3]. Creatine is primarily distributed in skeletal muscle as either free creatine or phosphocreatine [1, 4], where it functions as part of the phosphagen energy system to provide energy and facilitate ATP resynthesis via creatine kinase, in particular during very high intensity exercise [2, 5]. Creatine absorption is mediated by sodium and chloride-dependent transporters where it is first absorbed in small intestines, then distributed throughout the body, where skeletal muscle serves as a primary reserve [6, 7]. Exogenous creatine supplementation has been demonstrated to enhance intramuscular stores by 20–40% and elicit ergogenic effects [1–3, 8].

A 28–30 day creatine supplementation period has been shown to increase muscle creatine concentrations and improve exercise performance [9–13]. Short duration creatine loading phases (approximately 20 g of creatine for 5–7 days) have also been implemented, however, it has been demonstrated that 28 days of 3.0 g·d^− 1^ of creatine monohydrate supplementation resulted in approximately the same 20% increase in total muscle creatine [14]. Thus, 28 days of creatine supplementation is sufficient to examine the potential ergogenic effects.

Creatine monohydrate (CM) is the most common delivery form due to its high bioavailability and stability. However, numerous forms of creatine exist in the nutritional supplement market, such as creatine citrate, creatine hydrochloride, creatine pyruvate, creatine malate, and sodium creatine phosphate [2, 15]. Furthermore, the formulation of a salt-creatine complex functions to improve solubility in solution due to the reduction in pH from the salt’s acid moiety, as well as functioning to improve the stability of creatine in solution where a low pH reduces intramolecular cyclization that results in the degradation of creatine to creatinine [15]. Recent studies have demonstrated that these alternative formulations may improve bioavailability and exercise performance [9, 16]. Thus, while CM has been well established as an effective delivery form to elicit improvements in exercise performance, more research is warranted to examine whether other creatine formulations may exhibit similar, or potentially greater overall improvements in performance.

During exercise, the production of reactive oxygen species in the muscle has been associated with the development of muscular fatigue due to its detrimental effects on excitation-contraction coupling and gene expression, as well as general oxidative stress on intramuscular lipids, proteins, and DNA [17, 18]. It has been demonstrated that supplementation with antioxidants may attenuate the deleterious effects of reactive oxygen species produced from exercise, potentially resulting in faster muscle recovery [18–20]. Polyphenols are a family of plant-derived metabolites including stilbenes, lignans, phenolic acids, and flavonoids that function as antioxidants [20]. While evidence suggests polyphenol supplementation may improve exercise performance [19, 21], the efficacy of polyphenol supplementation on measures of muscular strength, power, and endurance remains to be fully elucidated. Therefore, the purpose of the present study was to compare the effects of 28 days of supplementation with a blend of phosphocreatine disodium salts plus blueberry extract (PCDSB), CM, and placebo on measures of muscular strength, power, and endurance. Based on the findings of previous studies [11, 22, 23], we hypothesized that supplementation with PCDSB would exhibit greater improvements in muscular strength, power, and endurance than supplementation with CM or placebo.

## Methods

### Experimental approach

The present study implemented a double-blind, placebo-controlled, parallel design. The study consisted of a familiarization visit, a pre-supplementation test visit, 28 days of daily supplementation, and a post-supplementation test visit. During the testing visits, the subjects performed the fatigue test of Thorstensson and Karlsson [24] which consisted of 50 maximal, unilateral, concentric, isokinetic leg extensions of the left leg at 180°·s^− 1^. After completion of the pre-supplementation test visit, the subjects were randomly assigned to a supplemental group of phosphocreatine disodium salts plus blueberry extract (PCDSB; *n* = 11), an active control of creatine monohydrate (CM; *n* = 12), or a placebo (*n* = 10). The supplements were designed to be as similar as possible in volume, color, and taste, and included 5.0 g of phosphocreatine disodium salts plus 200 mg of blueberry extract in the PCDSB, 3.0 g of creatine monohydrate in the CM, and microcrystalline cellulose in the placebo group. The 5.0 g of PCDSB was 50% pure creatine and, therefore, each dose of the PCDSB contained 2.5 g of pure creatine. The 3.0 g of CM was 80% pure creatine and contained 2.4 g of pure creatine per dose. The blueberry extract was prepared from *Vaccinium angustifolium* berries and was standardized to 30% total phenols using the Follin-C assay. Thus, the 200 mg of blueberry extract in the PCDSB included 60 mg of phenols which consisted of a majority of proanthocyanins and a minority of anthocyanins. The bulk powder mixtures were sieved through a 60-mesh sieve to ensure uniform particle size and to get rid of the agglomerates. They were further blended for 1 h using an Erweka V-mixer (Model AR 403, Erweka America Inc., Edison, NJ) prior to packaging. Homogeneity of mixing was visually confirmed with the coloring agents used. Blended powders were accurately weighed (mass ± 1%) into individual foil lined pouches using a calibrated balance (Model SX 410, Denver Instruments, Bohemia, NY) and heat sealed. Five quality control samples were randomly selected from the packaged batch throughout the packaging process to ensure even distribution of contents, verified by mass for all samples and for creatine content in samples containing either PCDSB or CM by HPLC analysis. The HPLC method used was previously developed and validated per USP standards for accuracy, precision, and detection/quantitation limits. Samples were run on a Waters Acquity H-Class UPLC (Waters Inc. Framingham, MA) equipped with a photodiode array, column oven, and refrigerated autosampler. Creatine and creatinine peaks were separated using a 250 mm × 4.6 mm 5 μm Agilent ZORBAX Eclipse XDB-C18 base deactivated C18 column with a mobile phase consisting of 0.05 M ammonium sulfate at a flow rate of 1.5 ml/min and a temperature of 60 °C. Detection of standards and samples was performed at 206 nm using a photodiode array. All of the ingredients utilized in this study were supplied by Phenolics LLC (Omaha, NE). The subjects were provided with 28 days of individually packaged packets of powder of their assigned supplement and were instructed to mix the contents of the packet with 8 oz. of water and drink it immediately after mixing. Twenty-eight days of supplementation was based on the findings of Hultman et al. [14] who reported that 3.0 g·d^− 1^ of CM for 28 days resulted in approximately the same 20% increase in muscle total creatine concentration as loading with 20 g·d^− 1^ for 6 days. The subjects were instructed to maintain their usual exercise and dietary habits throughout the study. Each subject completed a 3-day dietary recall form and a 7-day exercise recall form prior to the pre-supplementation testing visit and prior to the post-supplementation testing visit to determine if there were changes in total caloric and macronutrient (carbohydrate, protein, and fat) intake as well as exercise (aerobic and resistance training) time during the supplementation period.

### Subjects

Thirty-three men volunteered to participate in this study (Table 1). Prior to testing, the subjects were screened for any medications, dietary supplements, nutritional products, or dietary programs that would potentially confound the results of this study. Additionally, the subjects reported no cardiovascular, metabolic, pulmonary, or musculoskeletal diseases. All subjects signed a written Informed Consent and completed a Health History Questionnaire that were approved by the Institutional Review Board for Human Subjects at the University of Nebraska-Lincoln (IRB #20200319941FB).

**Table 1:** Mean ± SD of Subject Characteristics

	Creatine Monohydrate Group (*n* = 12)	Phosphocreatine Disodium Salts Plus Blueberry Extract Group (*n* = 11)	Placebo Group (*n* = 10)	Composite (*n* = 33)
Age (years)	19.8 ± 1.1	20.3 ± 0.9	20.6 ± 1.2	20.2 ± 1.1
Height (cm)	179.0 ± 4.0	183.5 ± 4.8	178.2 ± 6.7	180.3 ± 5.5
Body Mass (kg)				
Pre- Supplementation	85.7 ± 21.4	89.6 ± 12.6	80.8 ± 11.9	85.5 ± 16.1
Post-Supplementation	86.4 ± 21.1	89.8 ± 12.9	80.6 ± 11.7	85.8 ± 16.1

Note: There was no significant interaction (*p* = 0.433, η^2^_p_ = 0.054) or main effect for Time (*p* = 0.409, η^2^_p_ = 0.023) or Group (*p* = 0.450, η^2^_p_ = 0.052) for body mass across 28 days of supplementation

### Familiarization visit

The first visit served as an orientation for the subjects to become familiar with the testing procedures to be completed during the subsequent test visits. During this visit, the subjects performed submaximal and maximal isokinetic leg extensions of the left leg at 180°·s^− 1^ on a calibrated Cybex II isokinetic dynamometer (Lumex Inc., Bay Shore, NY, USA). At the end of the familiarization visit, the subjects were asked to complete a 3-day dietary recall form and a 7-day exercise recall form by the day of the subsequent test visit and to abstain from exercise 24 h prior to the test visit.

### Test visits

Prior to testing, all subjects returned their completed 3-day dietary recall form and 7-day exercise recall form. The subjects performed a standardized warm-up consisting of 5 min of cycle ergometry at 50 W (Ergomedic 828E, Monark, Varberg, Sweden). The subjects were then seated on the isokinetic dynamometer with the head of the dynamometer aligned with the axis of rotation of the left knee. The distal portion of the lever arm of the dynamometer was affixed to the left ankle with a Velcro strap according to the manufacturer’s recommendations [25]. The subjects then completed the fatigue test, consisting of 50 maximal, unilateral, concentric, isokinetic leg extensions at 180°·s^− 1^. The investigators provided verbal encouragement throughout the test. For all isokinetic repetitions in the present study, the damping of the Cybex II was set at 2.

### Supplementation

Following the pre-supplementation fatigue test, the subjects were provided with 28 days of individually packaged powder of their assigned supplement. Investigators contacted each subject on a weekly basis to promote adherence and to ensure that the subjects experienced no adverse or serious adverse events related to the supplementation protocol. Five days prior to completion of the supplementation period, investigators scheduled the subjects for their post-supplementation test visit, instructed the subjects to complete a second 3-day dietary recall form and 7-day exercise recall form before the test visit, and reminded the subjects to abstain from exercise 24 h prior to the test visit.

### Determination of peak torque, average power, and percent decline

For the pre-supplementation and post-supplementation test visits, peak torque (PT) and average power (AP) were assessed during the fatigue test. Peak torque and AP were defined as the mean values from the first three repetitions of the fatigue test [24]. Average power was operationally defined in the as the time averaged, integrated area under the isokinetic torque curve. To calculate the AP, the voltage-time relationships from the Cybex II strip chart output was converted to the isokinetic torque curve by converting the voltage to torque in N·m based on a regression equation developed from the Cybex II calibration procedures recommended by the manufacturer [25]. Integration of the area under the torque curve was accomplished using a custom LabVIEW program (National Instrument, Austin, TX, USA) and the AP was then calculated by dividing the integrated area under the torque curve by the time for each isokinetic repetition. Percent decline across the fatigue test were calculated for peak toque (PT%) and average power (AP%) as:

$$ Percent\ Decline=\left[\left( Mean\ of\ First\ 3\  Repetitions- Mean\ of\ Last\ 3\  Repetitions\right)\div Mean\ of\ First\ 3\  Repetitions\ \right]\times 100 $$

### Statistical analyses

Test-retest reliability for PT and AT for the placebo group were measured 28 days apart and assessed with a repeated measures ANOVA (2,1 model) to determine systematic error, the interclass correlation coefficient (ICC) and the standard error of measurement (SEM) [26]. Peak torque, AP, PT%, and AP% were analyzed using 3 (Group [PCDSB, CM, and placebo]) × 2 (Time [Pre-supplementation and post-supplementation]) mixed factorial ANOVAs and paired *t*-tests were used for a priori, planned comparisons across the 28 days of supplementation within each group [27]. To determine important changes in PT and AP for individual subjects, the minimal important difference (MID) values were calculated as [28]:

$$ MID={SD}_{pooled}\times 0.5 $$

Where SD_pooled_ was the pre-supplementation between-subjects standard deviation and 0.5 reflects a moderate effect size necessary to be considered an important change [28]. The proportion of subjects that exceeded the MID for PT, AT, PT%, and AP% were examined between each group using separate Chi-squared (χ^2^) tests. Total calories, carbohydrate, protein, and fat intake as well as aerobic exercise, resistance training exercise, and total exercise time from pre- and post-supplementation were assessed with separate 3 (Group [PCDSB, CM, and placebo]) × 2 (Time [Pre-supplementation and post-supplementation]) mixed factorial ANOVAs. Partial eta squared (η^2^_p_) and Cohen’s *d* were calculated for each repeated measures ANOVA and pairwise comparison, respectively, and an alpha of *p* < 0.05 was considered statistically significant. All statistical analyses were performed using IBM SPSS v 27 (Armonk, NY, USA).

## Results

### Test-retest reliability

The test-retest reliability for PT and AP measured pre- and post-supplementation for the placebo group (*n* = 10) were examined for mean differences (systematic error), ICCs, and SEM using the 2,1 model of Weir [26]. The results demonstrated no significant mean differences between pre-supplementation and post-supplementation for PT (99.82 ± 36.51 N·m vs 107.52 ± 44.11 N·m; *p* = 0.122, η^2^_p_ = 0.256) or AP (141.94 ± 51.92 W vs 150.79 ± 64.30 W; *p* = 0.279, η^2^_p_ = 0.129; Table 2).

**Table 2:** Test-retest reliability of peak torque and average power from the placebo group (*n* = 10)

Visit 1 (Mean ± SD)	Visit 2 (Mean ± SD)	Grand Mean	***p***-Value	ICC	ICC 95% CI	SEM	CV (%)
**Peak Torque (N·m)**							
99.82 ± 36.51	107.52 ± 44.11	103.67	0.122	0.964	0.846–0.911	9.77	9.42
**Average Power (Watts)**							
141.94 ± 51.92	150.79 ± 64.30	146.37	0.279	0.953	0.824–0.988	17.18	11.74

*CV (%)* coefficient of variation; *ICC* intraclass correlation coefficient; *ICC 95% CI* intraclass correlation coefficient 95% confidence interval; *p-value* type I error rate for the one-way repeated measures ANOVA with a 2,1 model to assess systematic error, *SEM* standard error of measurement

### Dietary recall, exercise recall, and adverse events

During the 28 days of supplementation, there were no adverse or serious adverse events and all subjects reported consuming all daily doses of their supplement. Analyses for the dietary intake demonstrated no significant interactions (*p* = 0.160–0.400, η^2^_p_ = 0.59–0.115) or main effects for Time (*p* = 0.108–0.0.604, η^2^_p_ = 0.009–0.084) or Group (*p* = 0.537–0.7889, η^2^_p_ = 0.016–0.038) for total calories, carbohydrate, fat, or protein intake before and after the 28-day supplementation (Table 3). Analyses for exercise participation demonstrated no significant interactions (*p* = 0.116–0.395, η^2^_p_ = 0.60–0.134) or main effects for Time (*p* = 0.435–0.676, η^2^_p_ = 0.006–0.020) or Group (*p* = 0.248–0.856, η^2^_p_ = 0.010–0.089) for aerobic and resistance training, or total exercise time from before to after the 28 days of supplementation (Table 3). Furthermore, there was no significant interaction (*p* = 0.433, η^2^_p_ = 0.054) or main effect for Time (*p* = 0.409, η^2^_p_ = 0.023) or Group (*p* = 0.450, η^2^_p_ = 0.052) for body mass across 28 days of supplementation (Table 1).

**Table 3:** Mean ± SD of dietary intake and exercise participation before and after 28 days of supplementation

Group	Pre- Supplementation	Post-Supplementation
**Dietary Intake**		
**Calories (kcals)**		
Creatine Monohydrate Group (*n* = 12)	1875.7 ± 656.2	1706.4 ± 581.5
Phosphocreatine Disodium Salts Plus Blueberry Extract Group (*n* = 11)	1704.8 ± 464.8	1781.7 ± 312.7
Placebo Group (*n* = 10)	1734.5 ± 377.3	1463.8 ± 223.4
**Carbohydrates (g)**		
Creatine Monohydrate Group	173.4 ± 73.5	173.4 ± 70.4
Phosphocreatine Disodium Salts Plus Blueberry Extract Group	154.5 ± 67.0	161.3 ± 42.0
Placebo Group	187.1 ± 61.4	150.1 ± 36.7
**Fats (g)**		
Creatine Monohydrate Group	73.6 ± 30.3	66.7 ± 25.2
Phosphocreatine Disodium Salts Plus Blueberry Extract Group	59.8 ± 23.0	66.0 ± 24.3
Placebo Group	64.6 ± 16.5	58.1 ± 18.4
**Protein (g)**		
Creatine Monohydrate Group	100.5 ± 52.1	94.6 ± 35.3
Phosphocreatine Disodium Salts Plus Blueberry Extract Group	100.0 ± 473.7	107.7 ± 33.1
Placebo Group	99.4 ± 24.5	87.6 ± 23.2
**Exercise Participation**		
**Aerobic Training (hours/week)**		
Creatine Monohydrate Group	2.2 ± 2.3	1.7 ± 2.3
Phosphocreatine Disodium Salts Plus Blueberry Extract Group	0.9 ± 1.6	1.0 ± 1.5
Placebo Group	2.5 ± 2.2	2.7 ± 3.0
**Resistance Training (hours/week)**		
Creatine Monohydrate Group	2.0 ± 1.6	3.4 ± 2.6
Phosphocreatine Disodium Salts Plus Blueberry Extract Group	3.2 ± 2.3	3.2 ± 2.1
Placebo Group	2.4 ± 1.7	2.0 ± 1.6
**Total Exercise (hours/week)**		
Creatine Monohydrate Group	4.2 ± 3.0	5.0 ± 3.4
Phosphocreatine Disodium Salts Plus Blueberry Extract Group	4.2 ± 1.6	4.2 ± 1.7
Placebo Group	4.9 ± 2.3	4.6 ± 3.2

Note: Note: Analyses for the dietary intake demonstrated no significant interactions (*p* = 0.160–0.400, η^2^_p_ = 0.59–0.115) or main effects for Time (*p* = 0.108–0.0.604, η^2^_p_ = 0.009–0.084) or Group (*p* = 0.537–0.7889, η^2^_p_ = 0.016–0.038) for total calories, carbohydrate, fat, or protein intake before and after the 28-day supplementation. Analyses for exercise participation also demonstrated no significant interactions (*p* = 0.116–0.395, η^2^_p_ = 0.60–0.134) or main effects for Time (*p* = 0.435–0.676, η^2^_p_ = 0.006–0.020) or Group (*p* = 0.248–0.856, η^2^_p_ = 0.010–0.089) for aerobic and resistance training, or total exercise time

### Peak torque and average power

The results of the mixed factorial ANOVA for PT indicated no significant interaction (*p* = 0.135, η^2^_p_ = 0.125), or main effect for Group (*p* = 0.900, η^2^_p_ = 0.007), but there was a significant main effect for Time (*p* < 0.001, η^2^_p_ = 0.520) where post-supplementation (113.04 ± 32.49 N·m) was significantly (*p* < 0.001, *d* = 0.44) greater than pre-supplementation (99.89 ± 26.58 N·m). Planned pairwise comparisons demonstrated significant increases in PT from pre- to post-supplementation for the PCDSB group (99.90 ± 22.47 N·m vs 119.22 ± 29.87 N·m, *p* < 0.001, *d* = 0.73) and the CM group (99.95 ± 22.50 N·m vs 111.97 ± 24.50 N·m; *p* = 0.009, *d* = 0.51), but not for the placebo group (99.82 ± 36.51 N·m vs 107.52 ± 44.11, *p* = 0.112, *d* = 0.19; Table 4).

**Table 4:** Mean ± SD of peak torque and average power before and after 28 days of supplementation

Group	Pre-Supplementation	Post-Supplementation	***p***-value	Change (%)
**Peak Torque (N·m)**				
Creatine Monohydrate Group (*n* = 12)	99.95 ± 22.50	111.97 ± 24.50*	0.009	10.47 ± 11.03
Phosphocreatine Disodium Salts Plus Blueberry Extract Group (*n* = 11)	99.90 ± 22.47	119.22 ± 29.87*	< 0.001	14.74 ± 9.37
Placebo Group (*n* = 10)	99.82 ± 36.51	107.52 ± 44.11	0.112	4.43 ± 17.29
**Average Power (Watts)**				
Creatine Monohydrate Group	143.42 ± 33.84	159.78 ± 31.20*	0.021	10.22 ± 13.62
Phosphocreatine Disodium Salts Plus Blueberry Extract Group	140.18 ± 32.08	170.12 ± 42.68*	< 0.001	15.56 ± 13.05
Placebo Group	141.94 ± 51.92	150.79 ± 64.30	0.279	1.72 ± 21.95

* *p* < 0.05, post-supplementation > pre-supplementation

The results of the mixed factorial ANOVA for AP indicated no significant interaction (*p* = 0.103, η^2^_p_ = 0.141), or main effect for Group (*p* = 0.891, η^2^_p_ = 0.008), but there was a significant main effect for Time (*p* < 0.001, η^2^_p_ = 0.428) where post-supplementation (160.51 ± 46.13 W) was significantly (*p* < 0.001, *d* = 0.44) greater than pre-supplementation (141.85 ± 38.41 W). Planned pairwise comparisons demonstrated significant increases in AP from pre- to post-supplementation for the PCDSB group (140.18 ± 32.08 W vs 170.12 ± 42.68 W, *p* < 0.001, *d* = 0.79) and the CM group (143.42 ± 33.84 W vs 159.78 ± 31.20 W, *p* = 0.021, *d* = 0.50), but not for the placebo group (141.94 ± 51.92 W vs 150.79 ± 64.30 W; *p* = 0.279, *d* = 0.15; Table 4).

### Percent change scores for peak torque and average power

The results of the mixed factorial ANOVA for pre- versus post-supplementation PT% and AP% during the fatigue test demonstrated no significant interactions (*p* = 0.270–0.432, η^2^_p_ = 0.054–0.084), main effects for Time (*p* = 0.080–0.251, η^2^_p_ = 0.044–0.099), or main effects for Group (*p* = 0.917–0.993, η^2^_p_ = 0.001–0.006). Planned pairwise comparisons demonstrated no significant (*p* > 0.05) differences between pre-supplementation and post-supplementation for any of the groups (Table 5).

**Table 5:** Mean ± SD of percent declines in peak torque and average power during the fatigue test before and after 28 days of supplementation

Group	Pre-Supplementation	Post-Supplementation	***p***-value
**Peak Torque (%)**			
Creatine Monohydrate Group (*n* = 12)	40.30 ± 12.11	38.94 ± 18.36	0.780
Phosphocreatine Disodium Salts Plus Blueberry Extract Group (*n* = 11)	33.25 ± 31.39	43.69 ± 38.57	0.126
Placebo Group (*n* = 10)	38.00 ± 18.53	39.69 ± 25.65	0.728
**Average Power (%)**			
Creatine Monohydrate	40.65 ± 12.25	45.91 ± 15.60	0.211
Phosphocreatine Disodium Salts Plus Blueberry Extract Group	35.41 ± 30.36	44.82 ± 42.38	0.104
Placebo	38.85 ± 18.55	39.19 ± 27.80	0.949

### Minimal important difference for peak torque and average power

The MID for PT was 13.29 N·m which was exceed by 8 of the 11 subjects (73%) for the PCDSB group, 7 of the 12 subjects (58%) for the CM group, and 2 of the 10 subjects (20%) for the placebo group (Table 6). The results of the χ^2^ analyses demonstrated that a significantly (*p* = 0.016) greater proportion of subjects exceeded the MID for the PCDSB group (73%) than the placebo group (20%). There was no difference, however, in the proportion of subjects exceeding the MID between the CM group and placebo group (*p* = 0.069) or the PCDSB group and the CM group (*p* = 0.469).

**Table 6:** Absolute change (post-supplementation – pre-supplementation) values for peak torque with identification of the individual subjects that exceeded the minimal important difference

Creatine Monohydrate Group	Peak Torque Change (N·m)	Phosphocreatine Disodium Salts Plus Blueberry Extract Group	Peak Torque Change (N·m)	Placebo Group	Peak Torque Change (N·m)
Subject 1	14.45^a^	1	21.94^a^	1	25.53^a^
2	27.50^a^	2	16.64^a^	2	25.61^a^
3	15.01^a^	3	−3.31	3	9.19
4	16.64^a^	4	8.00	4	−22.38
5	−6.34	5	34.63^a^	5	4.24
6	2.06	6	18.51^a^	6	9.27
7	16.64^a^	7	8.80	7	−3.62
8	35.38^a^	8	28.07^a^	8	7.14
9	0.00	9	15.27^a^	9	8.94
10	−8.86	10	26.29^a^	10	13.04
11	20.42^a^	11	37.67^a^		
12	11.36				
Proportion exceeding the minimal important difference	7 of 12 (58%)		8 of 11 (73%) ^b^		2 of 10 (20%)

^a^Indicates an increase in peak torque across the 28 days of supplementation that exceeded the minimal important difference (pooled pre-supplementation S.D. × 0.5 effect size = 13.29 N·m). ^b^ Indicates a significantly (*p* < 0.05) greater proportion of individual subjects that exceeded the minimal important difference than the placebo group as determined by χ^2^ analyses

The MID for AP was 19.21 W which was exceed by 7 of the 11 subjects (64%) for the PCDSB group, 8 of the 12 subjects (67%) for the CM group, and 4 of the 10 subjects (40%) for the placebo group (Table 7). The results of the χ^2^ analyses indicated that there were no significant differences (*p* > 0.05) in the proportion of subjects that exceeded the MID for any of the group comparisons.

**Table 7:** Absolute change (post-supplementation – pre-supplementation) values for average power with identification of the individual subjects that exceeded the minimal important difference

Creatine Monohydrate Group	Average Power Change (Watts)	Phosphocreatine Disodium Salts Plus Blueberry Extract Group	Average Power Change (Watts)	Placebo Group	Average Power Change (Watts)
Subject 1	20.17^a^	1	32.42^a^	1	40.02^a^
2	37.18^a^	2	14.97	2	37.06^a^
3	8.24	3	−9.32	3	22.53^a^
4	26.33^a^	4	7.44	4	−33.27
5	−27.68	5	53.58^a^	5	2.64
6	−0.16	6	29.08^a^	6	14.75
7	23.83^a^	7	10.43	7	−23.47
8	35.17^a^	8	40.69^a^	8	5.59
9	39.52^a^	9	45.09^a^	9	−3.55
10	−15.18	10	49.01^a^	10	26.23^a^
11	27.15^a^	11	55.94^a^		
12	21.76^a^				
Proportion exceeding the minimal important difference	8 of 12 (67%)		7 of 11 (64%)		4 of 10 (40%)

^a^Indicates an increase in average power across the 28 days of supplementation that exceeded the minimal important difference (pooled pre-supplementation S.D. × 0.5 effect size = 19.21 W)

The MID for a reduction in the PT% from pre-supplementation to post-supplementation was − 10.77% which was exceeded by 1 of the 11 subjects (9%) for the PCDSB group, 3 of the 12 subjects (25%) for the CM group, and 2 of the 10 (20%) of subjects for the placebo group (Table 8). The results of the χ^2^ analyses indicated that there were no significant differences (*p* > 0.05) in the proportion of subjects that exceeded the MID for any of the group comparisons.

**Table 8:** Differences in percent decline for peak torque (post-supplementation – pre-supplementation) during that fatigue test with identification of the individual subjects that exceeded the minimal important difference

Creatine Monohydrate Group	Peak Torque % Decline Change	Phosphocreatine Disodium Salts Plus Blueberry Extract Group	Peak Torque % Decline Change	Placebo Group	Peak Torque % Decline Change
1	0.11	1	8.58	1	4.24
2	1.97	2	9.56	2	12.63
3	0.91	3	−29.01^a^	3	11.90
4	4.07	4	9.55	4	−26.67^a^
5	−20.67^a^	5	16.40	5	−0.99
6	2.75	6	55.55	6	−10.81^a^
7	−38.48^a^	7	−1.11	7	9.30
8	27.34	8	29.39	8	−9.62
9	0.00	9	8.88	9	0.81
10	7.75	10	9.37	10	26.07
11	10.53	11	−2.38		
12	−12.62^a^				
Proportion exceeding the minimal important difference	3 of 12 (25%)		1 of 11 (9%)		2 of 10 (20%)

^a^Indicates a reduction in percent decline for peak torque that exceeded the minimal important difference (pooled pre-supplementation S.D. × 0.5 effect size = −10.77%)

The MID for an improvement in AP% from pre-supplementation to post-supplementation was − 10.51% which was exceeded by 1 of the 11 subjects (9%) for the PCDSB group, 1 of the 12 subjects (8%) for the CM group, and 3 of the 10 (30%) of subjects for the placebo group (Table 9). The results of the χ^2^ analyses indicated that there were no significant differences (*p* > 0.05) in the proportion of subjects that exceeded the MID for any of the group comparisons.

**Table 9:** Differences in percent decline for average power (post-supplementation – pre-supplementation) during that fatigue test with identification of the individual subjects that exceeded the minimal important difference

Creatine Monohydrate Group	Average Power % Decline Change	Phosphocreatine Disodium Salts Plus Blueberry Extract Group	Average Power % Decline Change	Placebo Group	Average Power % Decline Change
1	0.07	1	11.37	1	6.64
2	5.16	2	9.15	2	10.17
3	0.17	3	−38.13^a^	3	11.39
4	6.49	4	14.25	4	−31.44^a^
5	−24.55^a^	5	18.79	5	−2.65
6	1.77	6	10.74	6	−13.68^a^
7	14.64	7	3.93	7	11.36
8	27.96	8	30.52	8	−12.37^a^
9	23.68	9	21.32	9	−0.97
10	6.81	10	5.80	10	24.92
11	8.16	11	15.70		
12	−7.31				
Proportion exceeding the minimal important difference	1 of 12 (8%)		1 of 11 (9%)		3 of 10 (30%)

^a^Indicates a reduction in percent decline for average power that exceeded the minimal important difference (pooled pre-supplementation S.D. × 0.5 effect size = −10.51%)

## Discussion

The purpose of the present study was to compare the effects of 28 days of supplementation with PCDSB, CM, or placebo on measures of muscular strength, power, and endurance. The reliability analyses (Table 2) for the placebo group across the 28 days of supplementation indicated excellent reliability [29] for PT and AP with ICCs of R = 0.9464 and R = 0.953, respectively. There was no systematic error between the test and re-test assessments as indicated by no mean differences (*p* = 0.122–0.279) for PT or AP. These values were consistent with previous studies of test-retest reliability for unilateral, isokinetic leg extensions [30, 31]. The findings of the present study indicated that 28 days of supplementation with PCDSB and CM resulted in comparable increases in PT (14.74 ± 9.37% versus 10.47 ± 11.03%, respectively) and AP (15.56 ± 13.05% versus 10.22 ± 13.62%, respectively), but had no effects on fatigue-induced PT% and AP%. It is possible that the effects of PCDSB and CM supplementation on parameters associated with muscular endurance would have been better evaluated using several repeated bouts of the fatigue tasks [32–34].

Creatine monohydrate is the most studied delivery form for examining the effects of creatine supplementation on performance and served as the active control to compare to PCDSB in this study. Previous studies [9, 35] have reported that CM supplementation with 2.0–5.0 g·d^− 1^ for 28–30 days increased muscular strength, power, and endurance. For example, Herda et al. [9] reported that 30 days of CM supplementation at 5.0 g·d^− 1^ resulted in improvements for the countermovement vertical jump, bench press 1RM, leg press 1RM, and the number of repetitions to failure at 80% 1RM for bench press and leg press. Their results [9] demonstrated that the CM supplementation increased muscular power (vertical jump), strength (bench press and leg press 1RM) and muscular endurance (repetitions to failure). Furthermore, Brenner et al. [35] reported that CM supplementation with 2.0 g·d^− 1^ for 28 days increased bench press 1RM in women. Thus, the improvements in PT and AP for the CM group, which served as the active control group in the present study, were consistent with previous studies that have demonstrated improvements in muscular strength following 28–30 days of CM supplementation.

Few studies [22, 36, 37] have examined the effects of supplementation with phosphocreatine on exercise performance outcomes. Peeters et al. [37] reported that phosphocreatine supplementation with 20 g·d^− 1^ for 3 days followed by 10 g·d^− 1^ for the remainder of the 6 week supplementation period, along with a 4 day per week of progressive, periodized resistance training program, increased bench press 1RM by 8.8 kg, with no difference compared the CM supplementation (11.1 kg). Eckerson et al. [22] found that 6 days of supplementation with 5.0 g of CM plus 4.0 g of sodium and potassium phosphates plus 18 g of dextrose increased anaerobic work capacity from the critical power test by 23.8% at day 3 and 49.8% at day 6, but CM supplementation and the placebo did not. A subsequent 30 day trial of the same supplement administered daily, however, showed no change in anaerobic work capacity [36]. The results of the present study extended these findings [22, 29, 38] and suggested that phosphocreatine supplementation in the form of PCDSB may be as effective in eliciting improvements in muscular strength as CM supplementation.

The present study was designed to compare the effects of 28 days of supplementation with PCDSB to an active control of CM, and an inactive placebo of microcrystalline cellulose. The PCDSB and CM were manufactured to contain a very similar amount of pure creatine. The 5.0 g of PCDSB was 50% pure creatine and, therefore, each dose of the PCDSB contained 2.5 g of pure creatine. The 3.0 g of CM was 80% pure creatine and contained 2.4 g of pure creatine per dose. Phosphocreatine disodium salts were selected for use in the PCDSB, in part, because of its greater solubility than CM and phosphocreatine may increase the variety of delivery forms that can be developed. Additionally, phosphocreatine disodium salts may have a greater bioavailability compared to CM and may saturate creatine stores at a faster rate, resulting in faster adaptations to performance compared to CM supplementation. The PCDSB contained sodium and blueberry extract. The CM and placebo did not contain sodium or blueberry extract and the PCDSB is a disodium salt of phosphocreatine which contributes the sodium during its absorption in the gastrointestinal tract. Furthermore, sodium is an electrolyte that mediates creatine transportation and aids in absorption and utilization throughout the body [39, 40]. For example, Peral et al. [6] examined the effects of sodium and chloride on creatine absorption in vitro and estimated that two sodium molecules and one chloride molecule are necessary for creatine transport. Furthermore, Dai et al. [41] reported that increased sodium concentration in an extracellular fluid enhanced creatine uptake in vitro. Thus, the mechanisms proposed by Peral et al. [6] and Dai et al. [41] suggest that the sodium content of PCDSB may function to facilitate greater creatine transporter function, resulting in a greater creatine absorption.

The blueberry extract in the PCDSB is rich in polyphenols including proanthocyanins and anthocyanins which exhibit antioxidant [19] and anti-inflammatory [42] properties and enhanced blood flow [43]. Evidence regarding the use of antioxidants have reported equivocal reports, including studies suggesting that antioxidants may blunt cellular mechanisms associated with adaptation and recovery [44, 45]. Antioxidant supplementation, however, has been demonstrated to improve strength [23, 46] and power [47], as well as delay the effects of fatigue [23, 48] and enhance recovery following eccentrically induced muscle damage [49]. Thus, theoretically, the blend of phosphocreatine, blueberry extract, and sodium may have had synergistic effects that resulted in significant improvements in exercise performance.

Some individuals respond to creatine supplementation with increases in muscle phosphocreatine stores and strength, while others do not [50]. Greenhaff et al. [51] suggested that 20–30% of individuals who underwent 5 days of creatine loading with 20 g·d^− 1^ “did not respond” based on an increase in total creatine of less than 10 mmol·kg^− 1^ dry weight. Syrotuik and Bell [50] reported that “responders” increased incline leg press 1RM by 25.8 kg following 5 days of creatine loading with approximately a mean of 23 g·d^− 1^ (0.3 g·kg^− 1^·d^− 1^), while the non-responders increased only by 2.0 kg. In the present study, the responses of the individual subjects were judged based on an MID that corresponded to a standardized effect size of 0.5 times the pre-supplementation pooled standard deviation [28, 38, 52]. For PT (Table 6), the PCDSB group exhibited a greater proportion (73%) of individual subjects who demonstrated increases across the 28 days of supplementation that exceeded the MID than the placebo group (20%). The proportion of individual subjects that exceeded the MID values for the CM group for PT (58%), however, was not significantly greater than the placebo group. Thus, when compared to the placebo group, supplementation with the blend of PCDSB provided an advantage over CM for the PT responses of the individual subjects. There were no differences in the proportions of individual subjects in the PCDSB, CM, and placebo groups that exceeded the MID for AP (Table 7), PT% (Table 8), or AP% (Table 9). For PT% and AP%, the proportions of individual subjects that exceeded the MID was less than or equal to 30%.

The lack of adverse and serious adverse events during the course of this study for the PCDSB, CM, and placebo groups were consistent with the safety of creatine supplementation [2, 3]. Furthermore, the results indicated that there were no significant (*p* > 0.05) mean differences between the PCDSB, CM, and placebo groups or changes across the 28 days of supplementation for body mass, total caloric intake, macronutrient intake, weekly aerobic training, weekly resistance training, or total weekly exercise (Tables 1 and 3). Thus, the 28 days of supplementation with PCDSB or CM had no effect on body mass. These findings were consistent with a previous study by Camic et al. [11] who reported no change in body mass across a 28 day supplementation period with polyethylene glycosylated (PEG) creatine. A subsequent study by Camic et al. [12], however, reported a significant 1.1 to 1.7% increase in body mass across 28 days of PEG creatine supplementation and Herda et al. [9] reported significant 0.4–1.0 kg increases in body mass following 30 days of CM and PEG creatine supplementation. Furthermore, Eckerson et al. [36] found a significant 1.0 kg increase in body mass following 30 days of supplementation with a blend of creatine, sodium, potassium, and dextrose.

The mean values for the dietary intake parameters in the present study were similar to those from recent studies [23, 53], but somewhat lower than expected for this sample [54]. This was likely due to the systematic underreporting of nutritional intake from the dietary recall form [55]. None of the subjects in the present study were competitive athletes, and the 7-day exercise recall indicated that they were recreationally trained [56].

Limitations in the present study include that the subjects did not return used supplement packets, but provided verbal confirmation that all packets had been consumed. While there was no significant Group by Time interaction for the repeated measures ANOVAS for PT and AP, the study was designed with a priori planned comparisons across Time within each supplement group, as well as the examination of the individual responses to supplementation. No blood or urine was collected to examine direct or indirect changes in intramuscular creatine stores throughout the supplementation. It has previously been demonstrated, however, that supplementation with 3 g/day of creatine monohydrate for 28 days significantly increased muscle total creatine concentrations by 20 mmol [14]. Furthermore, the supplementation of 5 g/day is consistent with the recommendations of the International Society of Sports Nutrition position stand on effective creatine dosing strategies [2]. A limitation of the present study was that the PCDSB included a blend of phosphocreatine, blueberry extract, and sodium. Thus, the positive effects of PCDSB on muscular strength and power may have been due to the additive effects of these ingredients. It was not possible with this study design to determine the individual effects of phosphocreatine disodium salts or blueberry extract on the performance outcomes, or their relative importance to the effects of PCDSB. Furthermore, like Herda et al. [9], the subjects in the present study maintained their normal exercise habits throughout the 28 days of supplementation. This methodology has substantial ecological validity since consumers of products like CM and PCDSB typically have a wide variety of exercise habits. Additional control, however, could be accomplished by including a prescribed exercise program with the supplementation. Other aspects of the current study design that should be further examined include the inclusion of female subjects and supplementation with higher doses for shorter and/or longer durations.

## Conclusions

In summary, the result of the present study indicated that 28 days of supplementation with PCDSB and CM resulted in comparable mean increases in PT and AP. There were no differences, however, for PT% and AP% across the fatigue test from pre- to post-supplementation for the PCDSB, CM, or placebo group. Evaluation of individual responses showed that a larger proportion of subjects exhibited increases in PT from pre- to post-supplementation that were greater than the MID values for the PCDSB group than the placebo group. Overall, the current findings indicated that 28 days of supplementation with both PCDSB and CM resulted in increases in PT and AP, however, there were no mean differences between these groups as determined by repeated measures ANOVA.. The PCDSB, however, may have an advantage over CM when compared to the placebo group for the proportion of individual subjects that respond favorably to supplementation with meaningful increases in muscular strength.

## Data Availability

The dataset analyzed may become available from the corresponding author upon a reasonable request.

## Abbreviations

ANOVA: Analysis of variance
AP: Average power
AP%: Average power percent decline
CM: Creatine Monohydrate
MID: Minimal Important Difference
PCDSB: Phosphocreatine disodium salts plus blueberry extract
PT: Peak torque
PT%: Peak torque percent decline

## References

[1] Andres S, Ziegenhagen R, Trefflich I, Pevny S, Schultrich K, Braun H, Schänzer W, Hirsch-Ernst KI, Schäfer B, Lampen A (2017). Creatine and creatine forms intended for sports nutrition. Mol Nutr Food Res.

[2] Kreider RB, Kalman DS, Antonio J, Ziegenfuss TN, Wildman R, Collins R (2017). International Society of Sports Nutrition position stand: safety and efficacy of creatine supplementation in exercise, sport, and medicine. J Int Soc Sports Nutr.

[3] Antonio J, Candow DG, Forbes SC, Gualano B, Jagim AR, Kreider RB, Rawson ES, Smith-Ryan AE, VanDusseldorp TA, Willoughby DS, Ziegenfuss TN (2021). Common questions and misconceptions about creatine supplementation: what does the scientific evidence really show?. J Int Soc Sports Nutr.

[4] Mesa JLM, Ruiz JR, González-Gross MM, Gutiérrez Sáinz A, Castillo Garzón MJ (2002). Oral creatine supplementation and skeletal muscle metabolism in physical exercise. Sports Med Auckl NZ.

[5] Schlattner U, Klaus A, Ramirez Rios S, Guzun R, Kay L, Tokarska-Schlattner M (2016). Cellular compartmentation of energy metabolism: creatine kinase microcompartments and recruitment of B-type creatine kinase to specific subcellular sites. Amino Acids.

[6] Peral MJ, García-Delgado M, Calonge ML, Durán JM, De La Horra MC, Wallimann T (2002). Human, rat and chicken small intestinal Na+ − cl- -creatine transporter: functional, molecular characterization and localization. J Physiol.

[7] Persky AM, Brazeau GA, Hochhaus G (2003). Pharmacokinetics of the dietary supplement Creatine. Clin Pharmacokinet.

[8] Buford TW, Kreider RB, Stout JR, Greenwood M, Campbell B, Spano M (2007). International Society of Sports Nutrition position stand: creatine supplementation and exercise. J Int Soc Sports Nutr.

[9] Herda TJ, Beck TW, Ryan ED, Smith AE, Walter AA, Hartman MJ, Stout JR, Cramer JT (2009). Effects of creatine monohydrate and polyethylene glycosylated creatine supplementation on muscular strength, endurance, and power output. J Strength Cond Res..

[10] Kerksick CM, Wilborn CD, Campbell WI, Harvey TM, Marcello BM, Roberts MD, Parker AG, Byars AG, Greenwood LD, Almada AL, Kreider RB, Greenwood M (2009). The effects of Creatine monohydrate supplementation with and without D-Pinitol on resistance training adaptations. J Strength Cond Res..

[11] Camic CL, Hendrix CR, Housh TJ, Zuniga JM, Mielke M, Johnson GO, Schmidt RJ, Housh DJ (2010). The effects of polyethylene glycosylated Creatine supplementation on muscular strength and power. J Strength Cond Res..

[12] Camic CL, Housh TJ, Zuniga JM, Traylor DA, Bergstrom HC, Schmidt RJ, Johnson GO, Housh DJ (2014). The effects of polyethylene glycosylated Creatine supplementation on anaerobic performance measures and body composition. J Strength Cond Res..

[13] Wang CC, Fang CC, Lee YH, Yang MT, Chan KH. Effects of 4-week Creatine supplementation combined with complex training on muscle damage and sport performance. Nutrients. 2018;10(11). 10.3390/nu10111640. 10.3390/nu10111640 PMC6265971 30400221

[14] Hultman E, Soderlund K, Timmons JA, Cederblad G, Greenhaff PL (1996). Muscle creatine loading in men. J Appl Physiol.

[15] Jäger R, Purpura M, Shao A, Inoue T, Kreider RB (2011). Analysis of the efficacy, safety, and regulatory status of novel forms of creatine. Amino Acids.

[16] Jäger R, Harris RC, Purpura M, Francaux M (2007). Comparison of new forms of creatine in raising plasma creatine levels. J Int Soc Sports Nutr.

[17] Powers SK, Jackson MJ (2008). Exercise-induced oxidative stress: cellular mechanisms and impact on muscle force production. Physiol Rev.

[18] Reid MB (2016). Reactive oxygen species as agents of fatigue. Med Sci Sports Exerc.

[19] McLeay Y, Barnes MJ, Mundel T, Hurst SM, Hurst RD, Stannard SR (2012). Effect of New Zealand blueberry consumption on recovery from eccentric exercise-induced muscle damage. J Int Soc Sports Nutr..

[20] Bowtell J, Kelly V (2019). Fruit-derived polyphenol supplementation for athlete recovery and performance. Sports Med Auckl Nz.

[21] Kashi DS, Shabir A, Da Boit M, Bailey SJ, Higgins MF. The efficacy of administering fruit-derived polyphenols to improve health biomarkers, exercise performance and related physiological responses. Nutrients. 2019;11(10). 10.3390/nu11102389. 10.3390/nu11102389 PMC6836214 31591287

[22] Eckerson JM, Stout JR, Moore GA, Stone NJ, Iwan KA, Gebauer AN, Ginsberg R (2005). Effect of creatine phosphate supplementation on anaerobic working capacity and body weight after two and six days of loading in men and women. J Strength Cond Res..

[23] Anders JPV, Keller JL, Smith CM, Hill EC, Housh TJ, Schmidt RJ, Johnson GO (2020). The effects of Asparagus Racemosus supplementation plus 8 weeks of resistance training on muscular strength and endurance. J Funct Morphol Kinesiol.

[24] Thorstensson A, Karlsson J (1976). Fatiguability and fibre composition of human skeletal muscle. Acta Physiol Scand.

[25] Cybex Division of Lumex Inc (1983). Cybed II User Manual.

[26] Weir JP (2005). Quantifying test-retest reliability using the intraclass correlation coefficient and the SEM. J Strength Cond Res..

[27] Keppel G (1991). Design and analysis: a researcher’s handbook.

[28] Norman GR, Sloan JA, Wyrwich KW (2003). Interpretation of changes in health-related quality of life: the remarkable universality of half a standard deviation. Med Care.

[29] Cicchetti DV (1994). Guidelines, criteria, and rules of thumb for evaluating normed and standardized assessment instruments in psychology. Psychol Assess.

[30] Perrin DH (1993). Isokinetic Exercise and Assessment. Human Kinetics.

[31] Neltner TJ, Anders JPV, Keller JL, Smith RW, Housh TJ, Schmidt RJ, Johnson GO (2020). Ipsilateral and contralateral torque responses to bilateral and unilateral maximal, fatiguing, isokinetic leg extensions. Int J Kinesiol Sports Sci.

[32] Balsom B, Ekblom B, Soerlund K, Sjodin K, Hultman E (1993). Creatine supplementation and dynamic high-intensity intermittent exercise. Scand J Med Sci Sports.

[33] Gilliam JD, Hohzorn C, Martin D, Trimble MH (2000). Effect of oral creatine supplementation on isokinetic torque production. Med Sci Sports Exerc.

[34] Sarshin A, Fallahi V, Forbes SC, Rahimi A, Koozehchian MS, Candow DG, Kaviani M, khalifeh SN, Abdollahi V, Naderi A (2021). Short-term co-ingestion of creatine and sodium bicarbonate improves anaerobic performance in trained taekwondo athletes. J Int Soc Sports Nutr..

[35] Brenner M, Rankin JW, Sebolt D (2000). The effect of creatine supplementation during resistance training in women. J Strength Cond Res.

[36] Eckerson JM, Bull AA, Moore GA (2008). Effect of thirty days of Creatine supplementation with phosphate salts on anaerobic working capacity and body weight in men. J Strength Cond Res..

[37] Peeters BM, Lantz CD, Mayhew JL (1999). Effect of Oral Creatine monohydrate and Creatine phosphate supplementation on maximal strength indices, body composition, and blood pressure. J Strength Cond Res..

[38] Hopkins WG (2004). How to interpret changes in an athletic performance test. Sportscience..

[39] Schoch RD, Willoughby D, Greenwood M (2006). The regulation and expression of the Creatine transporter: a brief review of Creatine supplementation in humans and animals. J Int Soc Sports Nutr..

[40] Hummer E, Suprak DN, Buddhadev HH, Brilla L, San Juan JG (2019). Creatine electrolyte supplement improves anaerobic power and strength: a randomized double-blind control study. J Int Soc Sports Nutr..

[41] Dai W, Vinnakota S, Qian X, Kunze DL, Sarkar HK (1999). Molecular characterization of the human CRT-1 creatine transporter expressed in Xenopus oocytes. Arch Biochem Biophys.

[42] Park CH, Kwak YS, Seo HK, Kim HY (2018). Assessing the values of blueberries intake on exercise performance, TAS, and inflammatory factors. Iran J Public Health.

[43] Klimis-Zacas D, Kristo AS (2012). Wild blueberries (*Vaccinium angustifolium*): modulators of vascular function, structure, and metabolism. Emerging Trends in Dietary Components for Preventing and Combating Disease. American Chemical Society.

[44] Merry TL, Ristow M (2016). Do antioxidant supplements interfere with skeletal muscle adaptation to exercise training?. J Physiol.

[45] Ito N, Ruegg UT, Kudo A, Miyagoe-Suzuki Y, Takeda S (2013). Activation of calcium signaling through Trpv1 by nNOS and peroxynitrite as a key trigger of skeletal muscle hypertrophy. Nat Med.

[46] Levers K, Dalton R, Galvan E, Goodenough C, O’Connor A, Simbo S, Barringer N, Mertens-Talcott SU, Rasmussen C, Greenwood M, Riechman S, Crouse S, Kreider RB (2015). Effects of powdered Montmorency tart cherry supplementation on an acute bout of intense lower body strength exercise in resistance trained males. J Int Soc Sports Nutr..

[47] Lafay S, Jan C, Nardon K, Lemaire B, Ibarra A, Roller M, Houvenaeghel M, Juhel C, Cara L (2009). Grape extract improves antioxidant status and physical performance in elite male athletes. J Sports Sci Med.

[48] McKenna MJ, Medved I, Goodman CA, Brown MJ, Bjorksten AR, Murphy KT (2006). N-acetylcysteine attenuates the decline in muscle Na+,K+-pump activity and delays fatigue during prolonged exercise in humans. J Physiol.

[49] Shafat A, Butler P, Jensen RL, Donnelly AE (2004). Effects of dietary supplementation with vitamins C and E on muscle function during and after eccentric contractions in humans. Eur J Appl Physiol.

[50] Syrotuik DG, Bell GJ (2004). Acute creatine monohydrate supplementation: a descriptive physiological profile of responders vs. nonresponders. J Strength Cond Res..

[51] Greenhaff PL, Bodin K, Soderlund K, Hultman E (1994). Effect of oral creatine supplementation on skeletal muscle phosphocreatine resynthesis. Am J Phys.

[52] Ferreira ML, Herbert RD, Ferreira PH, Latimer J, Ostelo RW, Nascimento DP, Smeets RJ (2012). A critical review of methods used to determine the smallest worthwhile effect of interventions for low back pain. J Clin Epidemiol.

[53] Keller JL, Housh TJ, Hill EC, Smith CM, Schmidt RJ, Johnson GO (2019). The effects of Shilajit supplementation on fatigue-induced decreases in muscular strength and serum hydroxyproline levels. J Int Soc Sports Nutr..

[54] U.S. Department of Health and Human Services and U.S (2015). Department of Agriculture. 2015–2020 Dietary Guidelines for Americans.

[55] Program I of M (US) C on DRA in the W (2002). Food-Based Assessment of Dietary Intake. National Academies Press (US).

[56] American College of Sports Medicine, Riebe D, Ehrman JK, Liguori G, Magal M. ACSM's guidelines fo exercise testing and prescription. Tenth Edition. Philadelphia (PA): Wolters Kluwer; 2018.

